# Substance P- and Insulin-like Growth Factor 1-derived Tetrapeptides for Neurotrophic Keratopathy Related to Leprosy: A Clinical Trial

**DOI:** 10.1016/j.xops.2024.100634

**Published:** 2024-10-21

**Authors:** Shoko Kondo, Yoshiko Okano, Satoshi Iraha, Shoji Tokunaga

**Affiliations:** 1 Department of Ophthalmology, National Sanatorium Kikuchi Keifuen, Koshi, Japan; 2 Department of Ophthalmology, National Sanatorium Oshima Seishoen, Takamatsu, Japan; 3 Department of Ophthalmology, Kumamoto University School of Medicine, Kumamoto, Japan; 4 Medical Information Center, Kyushu University Hospital, Fukuoka, Japan

**Keywords:** Insulin-like growth factor 1, Leprosy, Neurotrophic keratopathy, Substance P, Tetrapeptides

## Abstract

**Purpose:**

Neurotrophic keratopathy is part of the leprosy sequelae and causes progressive deterioration of visual acuity. Although leprosy is bacteriologically curable, there is currently no efficient treatment. Eye drops containing tetrapeptides, phenylalanine-glycine-leucine-methionine-amide (FGLM-NH_2_) and serine-serine-serine-arginine (SSSR), derived from substance P and insulin-like growth factor 1, are clinically efficacious in the treatment of corneal epithelial disorders caused by neurotrophic keratopathy. To further investigate the effect of this treatment on leprosy sequalae, we evaluated the clinical efficacy of FGLM-NH_2_+SSSR eye drops for treating neurotrophic keratopathy.

**Design:**

Clinical trial: interventional, multicenter, exploratory, single-arm, before and after comparison.

**Participants:**

The eyes (12) of 11 patients, aged >60 years, were studied from 2 leprosy sanatoriums in Japan.

**Methods:**

Patients with neurotrophic keratopathy in leprosy sanatorium, specifically those with corneal perception of <40 mm, assessed by the Cochet-Bonnet corneal esthesiometer, and persistent corneal epithelial defects (PEDs) or corneal stromal thinning, or both, were included in this study. Those treated for infection in the acute phase were excluded from the study. Eye drops containing FGLM-NH_2_ 0.05% and SSSR 5 × 10^-6^% were administered 4 times daily for up to 3 months. Fluorescein staining and optical corneal sections were photographed using a slit lamp microscope at protocol-set intervals. Where possible, anterior segment OCT was performed before and after the intervention.

**Main Outcome Measures:**

The primary outcome measured was improvement in neurotrophic keratopathy. The patient was judged to have improved when ≥1 of the following criteria were met: (1) healing epithelial defects or (2) increased thickness in the thin area of the cornea. Secondary end points were visual acuity, subjective findings, and time to complete healing for a PED.

**Results:**

Neurotrophic keratopathy on epithelial defects or stromal thickness improved in 83.3% of the patients (90% confidence interval 56.2%–97.0%, *P* < 0.00001). The mean value of corrected visual acuity increased −0.16 by logarithm of the minimum angle of resolution. There were no adverse events reported in association with the treatment.

**Conclusions:**

We confirmed that FGLM-NH_2_+SSSR eye drops are effective for neurotrophic keratopathy without any adverse reaction in leprosy. These results should be disseminated to any parties who could need this information.

**Financial Disclosure(s):**

Proprietary or commercial disclosure may be found in the Footnotes and Disclosures at the end of this article.

Leprosy is a chronic infectious disease caused by *Mycobacterium leprae*, which predominantly attacks the skin and peripheral nerves.^1^^,^^2^ Although the word leprosy is used as a medical term in this article, it should be noted that the name Hansen's disease, derived from the name of the discoverer of the pathogenic bacterium, is recommended to avoid discriminatory connotations. There are currently >200 000 new cases of infection annually worldwide, mainly in emerging and developing countries.^2^ It is possible to completely treat this disease through multidrug therapy that was introduced by the World Health Organization in the 1990s. However, nerve paralysis worsens deformities due to muscle atrophy and contribute to functional impairments, either primary or secondary in any stage of the disease, even if leprosy is bacteriologically treated.^3^ Lagophthalmos due to facial paralysis and corneal hypoesthesia due to trigeminal neuropathy are well known, both of which often cause corneal disease and pose a threat to vision impairment,^4^ although few reports discuss this corneal disorder in leprosy with respect to neurotrophic keratopathy.^5^

Neurotrophic keratopathy is a rare intractable disease caused by damage to the trigeminal nerve at any level, from the brain to the nerve terminal within the cornea.^6^ Healing of the corneal epithelial defect is delayed, and clouding and melting of the corneal stroma occur, leading to perforation in severe cases. Causes of neurotrophic keratopathy include herpetic disease, diabetes, brain or ocular surgical procedures, medications used, and congenital diseases, 1 of which is leprosy.^7^ Conventional treatments such as the use of hyaluronic acid eye drops, use of autologous serum eye drops, protection with ointments, and protection with therapeutic contact lenses have been purely symptomatic. The only currently approved eye drop treatment is that with recombinant human nerve growth factor, which has a high therapeutic effect but is too expensive to administer to all patients.^6^

Nishida et al reported that insulin-like growth factor 1 (IGF-1) facilitated corneal epithelial migration in the presence of neurotransmitter substance P in cultured rabbit corneas.^8^ Furthermore, they reported that 2 peptides of 4 amino acids, each comprising a bioactive site, were capable of resurfacing normal epithelium without vascular invasion.^9^ These 2 tetrapeptides are the C-terminal phenylalanine-glycine-leucine-methionine-amide (FGLM-NH_2_) of substance P and amino acid sequence serine-serine-serine-arginine (SSSR) of the C domain of IGF-1.^9^ Nishida et al reported successful treatment of neurotrophic keratopathy with eye drops containing substance P plus IGF-1,^10^ FGLM-amide plus IGF-1,^11^^,^^12^^,^^13^ and FGLM-amide plus SSSR.^14^ In Japan, clinical trials (up to phase II), using FGLM-NH_2_+SSSR eye drops, were conducted for persistent corneal epithelial defects (PEDs) in patients with encouraging results. An increase in the number of enrolled patients was required to show significant efficacy for government approval as a prescription drug; however, the development of the eye drops was abandoned by the company because of profitability.^15^

Neurotrophic keratopathy is considered a rare disease with an estimated prevalence of 1.6/10 000.^16^ Further, corneal sensory impairment due to leprosy is observed in approximately 10% of the patients.^17^ Hansen's disease sanatorium in Japan, which currently has approximately 800 patients,^18^ has a large concentration of individuals with rare diseases. Although we have been working on the ocular sequelae of leprosy for many years, we have not been able to minimize visual loss associated with neurotrophic keratopathy.^5^ In Japan, even in bacteriologically treated patients who live long lives, subsequent complications, especially ocular complications and vision loss, remain a serious problem.^19^ Therefore, to maintain the quality of life of younger patients affected by leprosy worldwide, it is necessary to prevent the worsening of undesirable sequelae. The purpose of this study was to treat neurotrophic keratopathy due to the sequelae of leprosy with FGLM-NH_2_+SSSR peptide eye drops and validate its clinical efficacy and safety to ultimately implement treatment options within areas where leprosy is endemic. The null hypothesis is that the peptide eye drops have no effect on the prevention of the worsening undesirable sequelae, and the alternative hypothesis is that the eye drops show curable effects on neurotrophic keratopathy in a sequela of leprosy.

## Methods

This trial was approved by the Clinical Research Network Fukuoka Certified Review Board. Furthermore, approval was obtained from the ethics committee of each sanatorium.

Experimental details and consent were described in detail to all participants using an explanatory document approved by the Institutional Review Board, with signed consent.

This clinical trial complied with the Declaration of Helsinki and ministerial ordinance of Good Clinical Practice.

Clinical trial registration: Japan Registry of Clinical Trials as Primary Registries in the World Health Organization Registry Network. The registered number of the clinical trial plan is jRCTs071220053 (https://jrct.niph.go.jp).

Clinical equipment and analysis software: Slit lamp biomicroscope for clinical observation and image photo - Haag-Streit BQ900, Topcon SL-8Z; Digital process 3CCD camera for image photo - JFC Sales Plan SP-321, Sony 3CCD ExwaveHAD DXC-C33; Image filing system - KOWA-VK2, Topcon IMAGEnet 2000; anterior segment OCT (AS-OCT) for measurement of corneal thickness - NIDEK RS-3000 (Onboard image analysis software: NAVIS-EX); Cochet-Bonnet corneal esthesiometer - HANDAYA’s model; image analysis software for area and width measurement - Image-J (Wayne Rasband, National Institute of Health); and statistical analyses software - Stata 18.0 (Stata Corp College Station).

Site Management Organization: The incorporated nonprofit organization, Investigator Initiated Study Promotion Center (Tokyo, Japan).

### Study Design

This study was a multicenter, exploratory, single-arm, clinical trial that compared the outcomes before and after treatment with eye drops.

Study drug used: The Good Manufacturing Practice grade peptides were synthesized at the Chinese production site of CS Bio, Ltd. The purity assessed by high performance liquid chromatography was >95%, and endotoxin control concentration was <10EU/mg. FGLM-NH2 0.05% (1 mM) + SSSR 5 × 10^−6^% (100 nM) ophthalmic solution: concentration was determined based on previous research.^9^ We used FGLM-NH_2_・acetate 0.508 mg/mL and SSSR・2 acetate 0.0519 μg/mL. Doubled concentration solutions of each peptide were prepared using autoclaved calcium- and magnesium-free phosphate buffer saline (pH 7.4) and mixed in equal volumes. The completed peptide solution was sterilized by passing it through a Millipore membrane filter with a pore size of 0.22 μm. The preparations were conducted by Kikuchi Keifuen’s pharmacist, using aseptic techniques on a clean bench. The bottle of eye drops was stored in the refrigerator, and the expiry date was 1 week after opening.

### Participants

This study included patients with neurotrophic keratopathy who were admitted to the National Hansen's Disease Sanatorium in Japan.

Eligibility criteria were as follows: (1) patients admitted to sanatoriums; (2) PEDs or corneal stromal thinning, or both (PED is a surface defect of the corneal epithelium that does not heal for more than one week); (3) those with corneal perception of <40-mm nylon yarn length by the Cochet-Bonnet corneal esthesiometer; (4) cases in which written informed consent was obtained; and (5) the minimum age of 60, as all residents are >60 years old (no age maximum). No exclusions were made based on sex.

Exclusion criteria were as follows: (1) history of allergy to eye drops and (2) suffering from an acute systemic or ophthalmic infection.

### Interventions

Treatment protocol: study drug dosage and administration method: eye drops containing FGLM-NH_2_ and SSSR were instilled 4 times a day. Only 1 drop was used at each administration and administered continuously for up to 3 months. Medication was administered by nurses. Any previously used eye drops could be used in combination but would not be changed during the study period.

Protocol treatment discontinuation criteria included the following: (1) severe adverse events; (2) change in concomitant eye drops or concomitant therapy during protocol treatment; and (3) participant request.

Patient background information included the following: age, sex, leprosy type, age of onset/healing of leprosy, ophthalmic/systemic complications, medical history, and eye drops currently in use.

Baseline assessments included the following: visual acuity, presence or absence of lagophthalmos, corneal perception, anterior segment photography (fluorescein staining, slit lamp optical section), blood biochemical testing (liver function test, C-reactive protein measurement), and AS-OCT where possible. All investigators and experts engaged in the initial and follow-up assessments coordinated their efforts through online meetings and followed the same study protocol.

Corneal observation method: (1) the area of the planar corneal epithelial defect is stained with fluorescein, a slit lamp micrograph is taken, and the area on the image is calculated. A reduction in the epithelial defect area is interpreted as a response to the study drug but is not considered an improvement unless complete epithelialization is observed. (2) The corneal thickness is determined by taking an optical cross-sectional photograph using a slit lamp microscope and measuring the ratio of the thickness of the thin and healthy portions in the same image. In available examples of AS-OCT, the thickness on OCT images is compared before and at the end of the study.

Observation schedule:

➢ Screening tests and medical history confirmation conducted 1 week prior to obtaining consent.
➢ After baseline assessment, protocol treatment begins (day 1).
➢ Progress will be evaluated on days 3, 7, 14, 21, 28, 42, 56, 70, and 84 (±1 day). An interview regarding subjective symptoms and an image test using a slit lamp microscope will be administered at each follow-up.
➢ A visual acuity test and AS-OCT imaging will be performed at the end of the observation.

Image analysis: The anonymized image data were submitted to a data center installed within the Site Management Organization, before being sent to a subinvestigator in charge of image analysis who was not engaged in examining patients. The image data included the following: (1) area of the planar epithelial defect stained with fluorescein and (2) corneal thickness of the thin and normal parts measured using the image analysis software, Image-J. Anterior segment OCT images were assessed by the image analysis software NAVIS-EX installed in the device, and the total thickness and stromal thickness of the cornea were measured.

### Outcomes

*Primary outcome*: The primary outcome was “improvement in neurotrophic keratopathy.” Participants were considered to have improved when ≥1 of the following 2 end points were met.

1) Healing of PEDs: disappearance of the corneal epithelial defect was considered an improvement.
2) Increased corneal thickness in the thinned area: corneal thinning thickness was measured using 3 methods: the corneal thickness ratio between the thin and normal portions with optical cross-sectional photographs, the total corneal thickness, and the corneal stroma thickness in the thin part with OCT. The values before instillation and at the end of the observation were compared.

According to the Mackie’s grading system for neurotrophic keratopathy;^20^ a PED is equivalent to grade II, and corneal thinning is considered to be equivalent to grade III. Since it may be possible to prevent the exacerbation of neurotrophic keratopathy by restoring the barrier mechanism of the corneal epithelium, wound healing and stromal recovery were the primary end points observed.

*Secondary outcomes*: (1) visual acuity, (2) subjective findings, and (3) time to complete healing for the PED.

### Sample Size

Target case number: Ten at National Sanatorium Kikuchi Keifuen and 2 at National Sanatorium Oshima Seishoen.

Rationale for the study setting: The decrease in corneal sensitivity in leprosy is approximately 10%.^17^ As of the end of February 2022, the number of patients with corneal epithelial defects and corneal thinning due to neurotrophic keratopathy were approximately 20 of 155 residents at Kikuchi Keifuen and approximately 4 of 41 residents at Oshima Seishoen. Assuming a consent rate of 50%, the number of target cases was estimated to be 10 for Kikuchi Keifuen and 2 for Oshima Seishoen.

### Statistical Analyses

*Significance level of the test*: As this was an exploratory clinical study with a limited number of cases, statistical multiplicity adjustment and subgroup analyses, as well as impairment or imputation of missing values or interim analyses, were not performed. Because the number of cases was small, the analyses were performed without making any distinction between binocular or unilateral eyes. In this study, the significance level was set at a 2-sided *P* value of 10%, allowing for an increase in the α error to reduce the β error. Confidence intervals (CIs) were 2-sided 90% to maintain consistency with the significance level of the test.

*Number of recruited participants*: The natural course of neurotrophic keratopathy is known to worsen over time, and improvement is rarely expected. Indeed, the improvement achieved with conventional treatment was nearly 0%. In this study, the null proportion of improvement was assumed to be in the range of 10% to 20%. Previous studies validating this medication reported an efficacy of >80%;^13^^,^^14^ however, we expected a lower efficacy considering the older age of our participants. As such, the expected improvement proportion was set at 65% to 75%. In the condition of the abovementioned ranges in null and expected proportions, it is sufficient to assess the efficacy in ≥9 patients with a statistical power of ≥80%. Taking dropout during follow-up into account, the number of cases (eyes) to be registered in this study was set to 12.

*Analysis of the primary end point (improvement rate)*: We calculated the improvement rate as the number of cases judged to have improved divided by the number of eligible cases recruited. The 90% CI was calculated by the exact method assuming binomial distribution. A test of the improvement rate against the null rate was performed using the binomial test. Dropouts and missing data were included in the denominator of the improvement rate. The null proportion of improvement was set to 15% at the statistical tests, considering the results of past trials and background of the patients to be recruited.

In addition to the comprehensive improvement criteria, we conducted the following analyses. For each change in the corneal epithelial defect area and corneal thickness, we calculated the mean value and 90% CI, estimated the mean value and its 90% CI of the difference, and perform 1-sample *t* test. The significance level for the test was set at a 2-sided *P* value of 10%.

*Analyses of secondary end points*: (1) Visual acuity: visual acuity was analyzed using the logarithm of the minimum angle of resolution. Visual acuity at enrollment and termination was compared using the same method as the primary end point. (2) Subjective symptoms: descriptive analyses, including the median and interquartile range, of each measurement were performed. Mixed-effects ordered logistic regression was used to test whether subjective symptoms improved. In the regression model, the response variable was an ordinal variable in which ratings of subjective symptoms were arranged in descending order. The explanatory variable was the number of days since the start of treatment, and the level of the random intercepts was a patient. (3) Time for corneal epithelial defect to resurface: because of the small sample size, the descriptive statistics were not calculated, and the observed times were listed.

*Safety analysi*s: The occurrence of adverse events was be compiled for Safety Analysis Set. Grade (evaluation with Common Terminology Criteria for Adverse Events v5.0), severity, measure for study drugs, outcomes, and causal relationships were tabulated.

All statistical analyses were performed using statistical analysis software (Stata 18.0).

## Results

Patient registration began on September 14, 2022, with the first enrollment on October 28, 2022, and the last visit on December 27, 2022. The observation end date was April 20, 2023. The actual number of registered participants was 11 (12 eyes/11 persons). Patient background information is summarized in Table 1, and the anamnesis of systemic disease is shown in Table S2 (available at www.ophthalmologyscience.org). All patients had sensory and motor nerve paralysis throughout their bodies, with sequelae especially noted in their extremities. The target eyes included 3 (25.0%) eyes with PEDs, 7 (58.3%) with corneal stromal thinning, and 2 (16.7%) with both conditions. Information on complications, history, and treatment with concomitant eye drops in the target eyes is shown in Table S3 (available at www.ophthalmologyscience.org). Concomitant drugs were mainly used to treat lagophthalmos. A therapeutic contact lens was used for 1 eye.

**Table 1:** Patient Background

Patient Background (n = 11)	Value
Age, yrs	
Mean (interquartile range) [min, max]	86.0 (82–92) [77, 97]
Age at onset of leprosy, yrs	12.0 (10–15) [5, 16]
Age at which leprosy was cured, yrs∗	51.0 (34–53) [22, 58]
Sex	
Female, no. (%)	5 (45.5%)
Male, no. (%)	6 (54.5%)
Type of leprosy	
WHO classification	
MB, no. (%)	10 (90.9%)
PB	1 (9.1%)
Ridley–Jopling classification	
LL, no. (%)	6 (54.5%)
BL	4 (36.4%)
BT	1 (9.1%)

BL = borderline lepromatous; BT = borderline tuberculoid; LL = lepromatous type; max = maximum; MB = multibacillary; min = minimum; PB = paucibacillary; WHO = World Health Organization.

The WHO classifications used in the clinical field.

The Ridley–Jopling classifications used academically.

∗ n = 9, time of healing was unknown in 2 cases.

**Figure 1:**
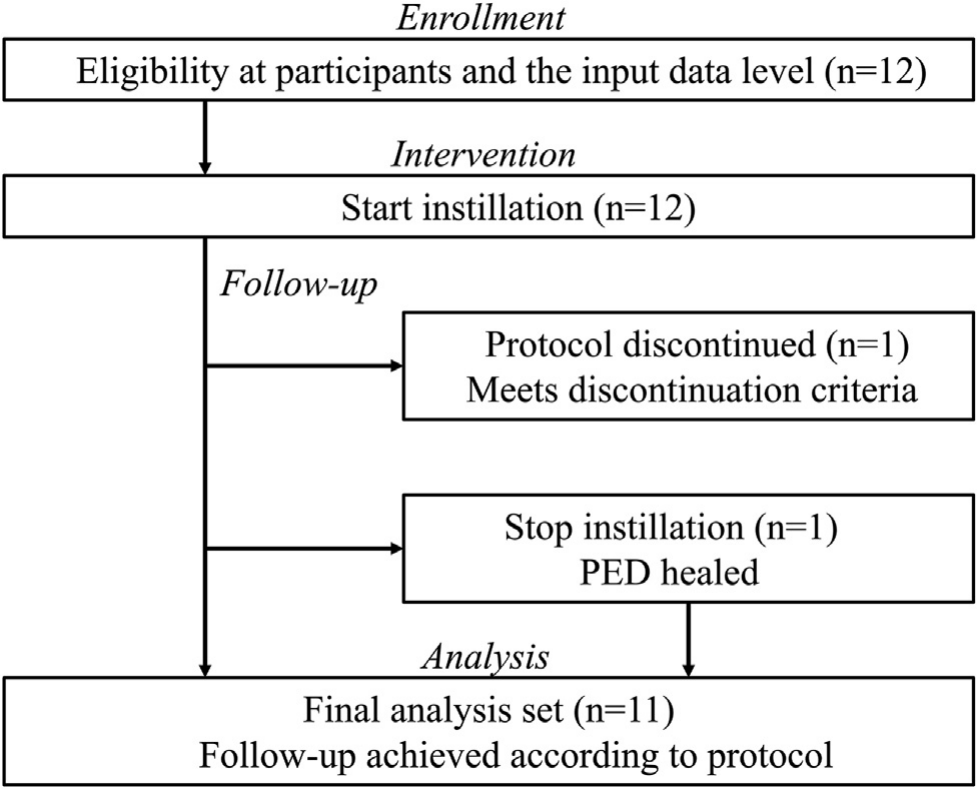
Flow diagram. PED = persistent corneal epitherial defect.

A flow diagram of patient recruitment procedure is shown in Figure 1. One participant met the discontinuation criteria on day 14. Because the eye developed a corneal ulcer after a routine examination for fundus disease at a specialized hospital, additional eye drops were administered. In another case, epithelial repair was observed from day 3, with complete disappearance of the epithelial defect by day 7. The eye drops were then continued for 1 week and were discontinued at the patient's request on day 14. Follow-up continued for 12 weeks, with no recurrence. The other 10 patients continued the treatment observation for 12 weeks.

### Primary End Point

The primary end point was improvement in neurotrophic keratopathy. The results are summarized in Table 4. The improvement rate for neurotrophic keratopathy (including discontinuation cases) was assessed and was calculated to be 83.3% (90% CI 56.2%–97.0%). The improvement rate was significant (*P* < 0.00001) with a null proportion of 15%.

**Table 4:** Primary Outcome: Determination Results of Improvement of Neurotrophic Keratopathy

Disease Name	Assessment of Injury Area			Sum (Number of Eyes)
	Improvements Available	Unchanged Deterioration	N/A (Canceled)	
Corneal epithelial disorders	3	0	0	3
Corneal stromal thinning	6	0	1	7
Corneal epithelial disorders and corneal stromal thinning	1∗	1†	0	2
Sum (number of eyes)	10	1	1	12

∗ Both the corneal epithelium and corneal thickness improved.

† The corneal epithelial defect did not improve, and the corneal thickness worsened. See Table S10 (available at www.ophthalmologyscience.org).

The respective changes in corneal epithelial defects and corneal thickness at enrollment and at the end of follow-up are explained below (Table 5):

1) Change in the corneal epithelial defect area: the corneal epithelial lesion area was reduced to 0 mm^2^ in 4 of 5 cases. The mean corneal epithelial defect area (90% CI) varied by −1.05 (−2.01, −0.06). The area of corneal epithelial damage was significantly decreased (*P* = 0.09) according to a 1-sample *t* test.
2) Changes in corneal thickness in areas of corneal thinning
  2.1. Corneal thickness ratio between the thin and normal portion (measurement of slit lamp optical cross-section photograph): optical sections are not necessarily vertical sections and cannot be compared with actual measurements. In the 2 cases with corneal opacity, accurate measurements could not be made. The changes in the corneal thickness ratio of 6 cases were analyzed. The mean corneal thickness ratio (90% CI) changed by 0.117 (0.063, 0.170). The corneal thickness ratio was significantly increased (*P* = 0.007) according to a 1-sample *t* test.
  2.2. Total corneal thickness in the thinned area (measured using OCT images): differences in the total corneal thickness at thinning sites in 8 cases were analyzed. The mean value (90% CI) of the total corneal thickness in the thinned region changed by 39.3 μm (5.7, 72.8). The total corneal thickness in the thinned area was significantly increased (*P* = 0.06) according to a 1-sample *t* test.
  2.3. Corneal stroma thickness in the thinned area (measured using OCT images): for 8 cases with corneal thinning, changes in the thickness of the corneal stroma at the thinning site were analyzed except for the effects of epithelium. The mean corneal stromal thickness (90% CI) in the thinning region varied by 24.5 μm (8.6, 40.4). The corneal stroma thickness in the thinned area was significantly increased (*P* = 0.02) according to a 1-sample *t* test.

**Table 5:** Breakdown of Primary Outcomes: Changes in the Area of Corneal Epithelial Defects and Corneal Thickness

Outcomes		Baseline	At the End of the Study	Difference (at the End of the Study—Baseline)
Corneal epithelial defect area (n = 5)				
Mean, mm^2^		1.27	0.22	−1.05
90% CI		(0.23, 2.31)	(−0.25, 0.69)	(−2.04, −0.06)
*P* value				0.09
Corneal thickness ratio between thin and normal portion (n = 6)∗				
Mean		0.187	0.303	0.117
90% CI		(0.080, 0.294)	(0.241, 0.365)	(0.063, 0.170)
*P* value				0.007
Total corneal thickness in the thinned area (n = 8)†				
Mean, μm		291.9	331.1	39.3
90% CI		(209.5, 374.2)	(258.0, 404.2)	(5.7, 72.8)
*P* value				0.06
Corneal stromal thickness in the thinned area (n = 8)†				
Mean, μm		203.3	227.8	24.5
90% CI		(117.9, 288.6)	(152.1, 303.4)	(8.6, 40.4)
*P* value				0.02

CI = confidence interval; n = number of eyes.

The *P* value was tested with a 1-sample *t* test.

∗ With optical section image.

† With anterior segment OCT image.

### Secondary End Points

(1) Visual acuity: the changes in corrected visual acuity of 11 cases were analyzed, and the results are shown in Table 6. The mean visual acuity (90% CI) changed by −0.16 (−0.29, −0.03). The change in visual acuity increased significantly from 0 (*P* = 0.05) according to a 1-sample *t* test.

**Table 6:** Secondary Outcomes: Visual Acuity

Corrected Visual Acuity (logMAR) (n = 11)	Baseline	At the End of the Study	Difference (at the End of the Study—Baseline)
Mean	1.30	1.14	−0.16
90% CI	(0.85, 1.75)	(0.69, 1.58)	(−0.29, −0.03)
*P* value			0.05

CI = confidence interval; logMAR = logarithm of the minimum angle of resolution; n = number of eyes.

The *P* value was tested with a 1-sample *t* test.

(2) Subjective symptoms: subjective symptoms were evaluated on a 5-point scale: 1 = bad, 2 = somewhat bad, 3 = fair, 4 = somewhat good, and 5 = good. These 5 levels are regarded as continuous quantities, and Table 7 shows the aggregation by evaluation period. On average, scores tended to get better with time. Mixed ordered logistic regression analysis estimated the odds ratio (90% CI) per week to be 1.18 (1.09, 1.29). This means that the probability that subjective symptoms increase by 1 unit per week is approximately 18%, and significant improvement in subjective symptoms was observed (*P* = 0.001).

**Table 7:** Evaluation of Subjective Symptoms as Continuous Amounts

Days since the Start of the Study	Baseline	Day 3	Day 7	Day 14	Day 21	Day 28	Day 42	Day 56	Day 70	Day 84
Number of eyes	12	12	12	12	10	10	10	10	10	10
Mean evaluation	2.3	2.8	3	2.8	2.4	2.7	3.5	2.9	3.2	3.5
SD	1.1	1.5	1	1.4	1.2	1.3	0.7	1.4	1	0.7

SD = standard deviation.

(3) Time taken for corneal epithelial defects to heal: the time taken for corneal epithelial defects to heal is expressed as the time from the start of treatment to the initial disappearance of the damaged epithelial area (Table 8). Five patients showed epithelial damage; in these patients, the area decreased to 0 within 84 days after treatment. However, it did not completely disappear in 1 patient. The number of days from the start of treatment until the corneal epithelial defect healed was 3, 7, 7, and 14 days in 4 cases and did not heal within 84 days in 1 case.

**Table 8:** Changes in the Corneal Epithelial Defect Area (mm^2^) and Time Taken to Heal

Corneal Epithelial Defect Area (mm^2^).										Time Taken to Heal (Days)
Baseline	Day 3	Day 7	Day 14	Day 21	Day 28	Day 42	Day 56	Day 70	Day 84	
0.26	0	0	0	3.54	0.19	0.19	0	0	0	3
0.96	1.14	0	0	0.05	0	0	0	0	0	7
0.39	0.34	0	0	.∗	.∗	.∗	.∗	.∗	0	7
2.84	0.51	0.83	0	0.87	0.78	1.05	0	0.25	0	14
1.91	1.71	1.91	2.53	2.13	1.43	1.35	0.34	1.24	1.1	－†

∗ Indicates a missing value.

† Corneal epithelial defect did not heal.

### Adverse Events

There was no adverse drug reactions observed. Table S9 (available at www.ophthalmologyscience.org) summarizes the adverse events in the Safety Analysis Set.

➢ Data for each case are included in the supplementary material, Table S10 (available at www.ophthalmologyscience.org).

## Discussion

The results of this study indicated that eye drops containing peptides derived from substance P and IGF-1 were effective in improving neurotrophic keratopathy in leprosy sequelae. With regard to the primary end points, PEDs lasted an average of 65.5 days (unpublished data that should have been collected at baseline, investigated from the medical record with consent) and were resurfaced completely in 4 of 5 eyes treated within 14 days after the initiation of treatment. Corneal thinning improved in 7 of 8 eyes by an average of 39.3 μm. The slight difference in the change in total corneal thickness and stroma thickness in the thin area was considered to be due to the recovery of other layers, including the epithelial layer. Regarding secondary end points, visual acuity and subjective symptoms showed improvement. As neurotrophic keratopathy does not heal spontaneously and tends to worsen over time, it is suggested that the current interventions are promising. Leprosy also causes a high rate of damage to the facial nerve, and 92% of the patients in this study had lagophthalmos. The research period was from November to April, which corresponds to the low-temperature period in Japan. In cases with lagophthalmos, the condition of the corneal surface often deteriorates during the cold and dry winter months. The 83.3% improvement over this period reinforces the evidence for the main outcomes.

As for the corneal epithelium, the pharmacological action of this agent lies in the effect of cell adhesion, which plays an important role in wound healing. Synergy between substance P and IGF-1 increases the expression of fibronectin receptors (integrins) in corneal epithelial cells, activates adhesion locale kinase and paxillin, which are local adhesion constituent molecules, and enhances the expression of the tight junction protein ZO-1 in epithelial cells.^7^ In 1 case in this study, which had a corneal epithelial defect that lasted for 3 months after cataract surgery, the epithelial defect healed 7 days after instillation, and healthy epithelium was maintained for 77 days until the end of the observation. People with a history of leprosy experience PEDs after cataract surgery, leading to corneal opacity and perforation and poor graft engraftment after corneal transplantation. It is generally known that corneal nerves are damaged after intraocular surgery.^21^ This drug has previously been clinically tested in a double-blind manner for corneal erosion after cataract surgery in patients with diabetes, and significant improvement was reported.^22^ This may help reduce corneal complications after eye surgery, which is a concern for patients with neurotrophic keratopathy.

In terms of corneal thickness, it is interesting to note that the test drug showed a significant increase in corneal stroma thickness. It is unclear whether the restoration of the barrier mechanism of the corneal epithelium by the research drug will indirectly help the corneal stroma to self-heal or whether this research drug will also directly work on the regeneration of the corneal stroma. Recently, it was reported that substance P promotes collagen synthesis by transforming growth factor-β and interleukin-1b in human corneal fibroblasts in vitro.^23^ For corneal stroma with nerve palsy, the test drug containing the bioactive site of substance P may have contributed to an increase in the stromal thickness due to collagen synthesis. Since some of our sanatoriums do not have AS-OCT equipment, the thickness of the cornea was also evaluated by optical section photographs of slit lamps to conduct this multi-institutional collaborative research. However, there were cases where the associate researcher in charge of image analysis was not able to measure accurately on the images, so the results of AS-OCT were emphasized. Anterior segment OCT, along with in vivo confocal microscopy, has been useful in determining the severity classification and treatment criteria for neurotrophic keratopathy^24^ and may be indispensable for the evaluation of corneal stroma.

In 1 case, where there was no improvement in the primary end point, debris was constantly deposited in a thin, deep epithelial defect, and the epithelial deletion was confirmed by OCT. In order to treat this patient, it was considered necessary to perform ulcer bottom scraping.

The average age of participants in the study was 86.0 years, representing the age of about 810 people currently in all leprosy sanatoriums in Japan.^18^ Neurotrophic keratopathy is thought to exist among recovered people living outside sanatoriums, but it is difficult to determine the number of neurotrophic keratopathy patients outside sanatoriums, as many of them hide the fact that they had contracted leprosy. In recent years, there have been <5 annual new cases of leprosy in Japan, most of them in young foreign workers from countries where leprosy is endemic.^18^ In addition, there are cases in which the patient has relapsed despite receiving multidrug therapy in their home country. For young patients with leprosy worldwide, it is important to detect and prevent the deterioration of neurotrophic keratopathy, which can be treated bacterially but threaten vision as they age. Currently, direct treatment of corneal neurotization such as autologous nerve transplantation or nerve growth factor is possible for neurotrophic keratopathy.^7^ However, it is difficult for everyone to avail these treatments. Considering the regionality of leprosy, it is hoped that the treatment of neurotrophic keratopathy will be somewhat affordable.

Because this study had a small sample size and was a single-arm before-after comparative study, it is difficult to conclude the effectiveness of therapeutic drugs based only on this outcome. Thus, randomized studies with larger sample sizes are needed for this drug to be better evaluated.

However, we confirmed that FGLM-NH_2_+SSSR eye drops are effective for neurotrophic keratopathy in the sequela of leprosy, without any adverse drug reactions. These results provide us with the rationale for using this drug as an in-hospital preparation for treatment. We expect that FGLM-NH_2_+SSSR eye drops will become an easily accessible treatment option to treat neurotrophic keratopathy for those needing it, whether doctors or patients.
