# Supplementary material for: Substance P- and Insulin-like Growth Factor 1-derived Tetrapeptides for Neurotrophic Keratopathy Related to Leprosy: A Clinical Trial
Source: Ophthalmol Sci. 2024 Oct 21;5(2):100634. doi: 10.1016/j.xops.2024.100634 (PMC11665617; doi:10.1016/j.xops.2024.100634)
Supplement: Table S10 [file mmc1.pdf]

Table S10. Patient Data

Image measurements were performed by personnel who had not examined the patients. The corneal thickness of the affected area could not be measured in some split lamp photographs.

KO-02L and KO-02R are the left and right eyes of the same patients.

KO-03L was discontinued on Day 14 due to the addition of a new eye drop.

In KO-05, the patient requested that eye drops be discontinued on Day 14 because PED had been cured. Follow-up observation according to the protocol was continued until Day 84.

PED: persistent corneal epithelial defect

ID	Age	Disease type	Epithelial defect area (mm²)			Ratio of corneal thickness between the thin and normal areas*		Total corneal thickness at its thin portion(μm) †		Corneal stromal thickness at its thin portion(μm) †		Visual acuity(logMAR)		Subjective symptoms ‡		Other key complications
			Base line	End of study	Days to healing	Baseline	End of study	Baseline	End of study	Baseline	End of study	Baseli ne	End of study	Basel ine	End of study	
KO-01R	77	Corneal thinning				0.44	0.43	385	372	237	252	2.0	2.0	1	4	Lagophthalmos
KO-02L	92	Corneal thinning				0.14	0.29	170	190	76	86	2.0	1.5	2	3	Lagophthalmos
KO-02R	92	Corneal thinning				0.09	0.21	160	256	82	141	1.5	1.4	2	3	Lagophthalmos
KO-03L	86	Corneal thinning				Unmeasurable	Dropout	No inspection	Dropout	No inspection	Dropout	1.7	Dropout	1	Dropout	Lagophthalmos
KO-04L	97	PED + Corneal thinning	0.96	0.00	7	Unmeasurable	Unmeasurable	194	327	121	169	2.9	2.9	1	3	Adhesive leucoma
KO-05L	80	PED	0.39	0.00	7							0.2	0.1	3	Missing	Lagophthalmos After cataract surgery
KO-06L	82	Corneal thinning				0.10	0.25	327	327	240	243	1.5	1.1	4	5	Lagophthalmos
KO-07L	92	PED	0.26	0.00	3							0.8	0.5	2	4	Lagophthalmos
KO-08R	82	Corneal thinning				0.20	0.32	419	442	287	320	0.5	0.7	4	4	Lagophthalmos
KO-09R	95	Corneal thinning				0.15	0.32	211	224	132	170	1.5	1.3	3	3	Lagophthalmos
KO-10R	86	PED	2.84	0.00	14							1.0	0.5	2	3	Lagophthalmos
KO-11L	89	PED + Corneal thinning	1.91	1.10	Not cured	Unmeasurable	Unmeasurable	469	511	451	441	0.4	0.5	2	3	Lagophthalmos

* Optical sections with slit lamp photography

† AS-OCT image analysis

‡ Subjective symptoms: 1=bad, 2=somewhat bad, 3=fair, 4=somewhat good, 5=good
