# Supplementary material for: Substance P- and Insulin-like Growth Factor 1-derived Tetrapeptides for Neurotrophic Keratopathy Related to Leprosy: A Clinical Trial
Source: Ophthalmol Sci. 2024 Oct 21;5(2):100634. doi: 10.1016/j.xops.2024.100634 (PMC11665617; doi:10.1016/j.xops.2024.100634)
Supplement: Table S2 [file mmc2.pdf]

Table S2. Systemic complications and medical history (n=11)

Systemic complications and medical history	Number of people	Percentage of participants, %
Drop foot	5	45.5
Knee osteoarthritis	5	45.5
Hypertension	5	45.5
Spinal stenosis (waist and chest)	5	45.5
Drop hand	3	27.3
Gastroesophageal reflux disease (GERD)	3	27.3
Cerebral infarction	3	27.3
Hyperlipidemia	3	27.3
Lower leg amputation	3	27.3
Fracture of femoral neck	2	18.2
Trigeminal neuralgia	2	18.2
Cervical spondylosis	2	18.2
Herpes zoster	2	18.2
Fracture of thoracic vertebrae	2	18.2
Osteoporosis	2	18.2
Coronary spastic angina	2	18.2
Hip osteoarthritis artificial joint	2	18.2
Ankle Charcot joint	2	18.2
Shoulder cuff injury	2	18.2
Insomnia	2	18.2
Complete right bundle branch block	1	9.1
Complete left bundle branch block	1	9.1
Supraventricular extrasystole	1	9.1
Femoral head necrosis	1	9.1
Tibia fracture	1	9.1
Calcaneus fracture	1	9.1
Hypothyroidism	1	9.1
Shoulder calcific tendinitis	1	9.1
Mild cognitive impairment	1	9.1
diabetes	1	9.1
Benign prostatic hyperplasia	1	9.1
Thoracic yellow ligament ossification	1	9.1
Chronic hepatitis C	1	9.1
Neurofibromatosis	1	9.1
Atomic bomb exposure	1	9.1
Cholelithiasis	1	9.1
Posterior peritoneal tumors	1	9.1
Hemiparesis	1	9.1
Fracture of left patella	1	9.1
Lumbar compression fracture	1	9.1
Deforming spondylosis	1	9.1
Cerebrospinal fluid hypovolemia	1	9.1
Severe depression	1	9.1
Generalized anxiety disorder	1	9.1
Parkinson's disease	1	9.1
Chronic obstructive pulmonary disease	1	9.1
Bronchial asthma	1	9.1
Hiatal hernia	1	9.1
Gluteal pressure ulcers	1	9.1
Lower leg amputation skin ulcer	1	9.1
prostate cancer	1	9.1
Gastrointestinal bleeding	1	9.1
Dysphagia	1	9.1
Dyslipidemia	1	9.1
Epistaxis	1	9.1

There were many cases of finger deformity and paralysis, although we could not include the names of the diseases.
