# Supplementary material for: Substance P- and Insulin-like Growth Factor 1-derived Tetrapeptides for Neurotrophic Keratopathy Related to Leprosy: A Clinical Trial
Source: Ophthalmol Sci. 2024 Oct 21;5(2):100634. doi: 10.1016/j.xops.2024.100634 (PMC11665617; doi:10.1016/j.xops.2024.100634)
Supplement: Table S3 [file mmc3.pdf]

Table S3. Background of the experimental eyes (n=12)

Background	Number of eyes	Percentage of target eyes, %
Current disease name		
Neurotrophic keratopathy	12	100.0
Facial nerve paralysis	11	91.7
Lagophthalmos keratopathy	10	83.3
Intraocular lens inserted eye	7	58.3
Aphakic eye	2	16.7
Chronic dacryocystitis	2	16.7
Nuclear cataract	1	8.3
Concomitant cataract	1	8.3
Postoperative persistent corneal epithelial defect	1	8.3
Band shaped keratopathy	1	8.3
Adhesive corneal vitiligo	1	8.3
After pseudo-pterygium surgery	1	8.3
Iridescent atrophy	1	8.3
Multiple pupils	1	8.3
Age-related macular degeneration	1	8.3
Trichiasis	1	8.3
Trigeminal neuralgia	1	8.3
Past history of the eyes		
Iridocyclitis (iritis)	9	75.0
Cataract (aging/concomitant) surgery	6	50.0
Lower eyelid auricular cartilage grafting	3	25.0
Corneal ulcer	3	25.0
Corneal perforation	2	16.7
Corneal mycosis	1	8.3
Allergic conjunctivitis	1	8.3
Traumatic scleral rupture (vitreous surgery)	1	8.3
Pterygium surgery	1	8.3
Lagophthalmos surgery (temporal muscle tendon	1	8.3
Ophthalmic eye drops currently in use		
Purified sodium hyaluronate 0.1%	12	100.0
White Vaseline	9	75.0
Saline	9	75.0
Pranoprofen	4	33.3
Iodine and polyvinyl alcohol	4	33.3
Artificial tears	3	25.0
Fluorometholone 0.02%	3	25.0
Ofloxacin eye ointment	2	16.7
Fradiomycin sulfate and methylprednisolone eye ointment	1	8.3
Diclofenac sodium	1	8.3
Rebamipide	1	8.3
Betamethasone sodium phosphate	1	8.3
Levofloxacin hydrate	1	8.3
Prednisolone acetate ester eye ointment	1	8.3
