# Supplementary material for: Substance P- and Insulin-like Growth Factor 1-derived Tetrapeptides for Neurotrophic Keratopathy Related to Leprosy: A Clinical Trial
Source: Ophthalmol Sci. 2024 Oct 21;5(2):100634. doi: 10.1016/j.xops.2024.100634 (PMC11665617; doi:10.1016/j.xops.2024.100634)
Supplement: Table S9 [file mmc4.pdf]

Table S9. Adverse events in SAS*

Event name	Overall number	(%)	Grade†			Severity		Measures‡		Outcome			Causation		
			1	2	3	Not severe	Severe	No change	Not applicable	Recovery	Remission	Unrecovered	No	Un-known	Yes
Upper respiratory tract infection	1	(2.6)	1			1		1		1			1		
Right heart failure	2	(5.3)		2		2		2				2	2		
Complete atrioventricular block	2	(5.3)			2		2	2		2			2		
Heart failure	2	(5.3)			2		2	2		2			2		
Corneal ulcer	1	(2.6)			1	1		1		1				1	
Infections and infestations, others															
Cellulitis at the indwelling site	2	(5.3)		2		2		2			2		2		
Skin and subcutaneous tissue disorders, others															
Tape rash	1	(2.6)	1			1		1		1			1		
Eye disorders, others															
Upper eyelid redness	1	(2.6)	1			1		1		1			1		
Dry lower eyelid margin	1	(2.6)	1			1			1			1	1		
Bulbar conjunctival hyperemia	1	(2.6)	1			1		1			1		1		
Increased hyperemia	1	(2.6)	1			1		1		1			1		
Hyperemia	3	(7.9)	3			3		3			2	1	3		
Corneal epithelial dryness	1	(2.6)	1			1		1		1			1		
New corneal erosion	1	(2.6)	1			1		1		1			1		
Secretion adhesion on contact lens	4	(10.5)	4			4		4		4				4	
Punctate erosion	2	(5.3)	2			2		1	1	1	1		2		
Superficial punctate keratopathy	4	(10.5)	4			4		4		4			4		
Trichiasis	1	(2.6)	1			1		1		1			1		
Filamentous keratopathy	2	(5.3)	2			2		1		2			2		
Conjunctival hyperemia	2	(5.3)	2			2		1	1	1	1		2		
Epithelial opacity at the center of the cornea	1	(2.6)	1			1		1			1			1	
Shallow erosion on the center of the cornea	1	(2.6)	1			1		1			1			1	
Mucus adhesion and punctate erosion on the center of the cornea	1	(2.6)	1			1		1		1			1		
Overall (%)	38	(100)	29 (76.3)	4 (10.5)	5 (13.2)	34 (89.5)	4 (10.5)	34 (91.9)	3 (8.1)	25 (65.8)	9 (23.7)	4 (10.5)	31 (81.6)	7 (18.4)	0 (0.0)

*Safety Analysis Set

†Evaluation by CTCAE v5.0 (Common Terminology Criteria for Adverse Events)

‡ Measures to be taken after the occurrence of adverse events
